# Effects of an intravenous ketamine infusion on inflammatory cytokine levels in male and female Sprague–Dawley rats

**DOI:** 10.1186/s12974-022-02434-w

**Published:** 2022-04-04

**Authors:** Haley F. Spencer, Rina Y. Berman, Martin Boese, Michael Zhang, Sharon Y. Kim, Kennett D. Radford, Kwang H. Choi

**Affiliations:** 1 Program in Neuroscience, Uniformed Services University, 4301 Jones Bridge Road, Bethesda, MD 20814 USA; 2 Center for the Study of Traumatic Stress, Uniformed Services University, 4301 Jones Bridge Road, Bethesda, MD 20814 USA; 3 Daniel K. Inouye Graduate School of Nursing, Uniformed Services University, 4301 Jones Bridge Road, Bethesda, MD 20814 USA; 4 Department of Psychiatry, Uniformed Services University, 4301 Jones Bridge Road, Bethesda, MD 20814 USA

## Abstract

**Background:**

Ketamine, a multimodal dissociative anesthetic drug, is widely used as an analgesic following traumatic injury. Although ketamine may produce anti-inflammatory effects when administered after injury, the immunomodulatory properties of intravenous (IV) ketamine in a non-inflammatory condition are unclear. In addition, most preclinical studies use an intraperitoneal (IP) injection of ketamine, which limits its clinical translation as patients usually receive an IV ketamine infusion after injury.

**Methods:**

Here, we administered sub-anesthetic doses of a single IV ketamine infusion (0, 10, or 40 mg/kg) to male and female Sprague–Dawley rats over a 2-h period. We collected blood samples at 2- and 4-h post-ketamine infusion to determine plasma inflammatory cytokine levels using multiplex immunoassays.

**Results:**

The 10 mg/kg ketamine infusion reduced spontaneous locomotor activity in male and female rats, while the 40 mg/kg infusion stimulated activity in female, but not male, rats. The IV ketamine infusion produced dose-dependent and sex-specific effects on plasma inflammatory cytokine levels. A ketamine infusion reduced KC/GRO and tumor necrosis factor alpha (TNF-α) levels in both male and female rats, interleukin-6 (IL-6) levels in female rats, and interleukin-10 (IL-10) levels in male rats. However, most cytokine levels returned to control levels at 4-h post-infusion, except for IL-6 levels in male rats and TNF-α levels in female rats, indicating a different trajectory of certain cytokine changes over time following ketamine administration.

**Conclusions:**

The current findings suggest that sub-anesthetic doses of an IV ketamine infusion may produce sex-related differences in the effects on peripheral inflammatory markers in rodents, and further research is warranted to determine potential therapeutic effects of an IV ketamine infusion in an inflammatory condition.

## Introduction

Ketamine is a multimodal anesthetic that is commonly used to produce analgesic relief for moderate to severe pain in service members following traumatic injuries [1, 2]. Ketamine is highly favorable for patients who are in hypovolemic shock due to its ability to preserve respiration and hemodynamic stability compared to other sedatives [1]. Thus, the United States Department of Defense Committee on Tactical Combat Casualty Care (TCCC) recommends the use of low-dose intravenous (IV) ketamine to induce analgesia, circumventing dissociative side effects produced at higher doses [3]. Alongside ketamine’s analgesic properties and high therapeutic index, a growing body of evidence has also demonstrated anti-inflammatory effects of ketamine in humans and rodents [4–8]. In addition, the long-held notion that ketamine should be contraindicated in traumatic brain injury (TBI) patients due to purported consequences of increased intracranial pressure has been disputed [9]. In fact, there is now growing interest in the therapeutic potential of ketamine following TBI given the drug’s neuroprotective and anti-inflammatory properties [10–12].

Early observations of ketamine administered to septic patients and animals [13, 14] heightened interest in characterizing ketamine’s immunomodulatory properties. This led to laboratory investigations which have demonstrated peripheral and central actions of ketamine on the immune system [7, 8]. Interestingly, ketamine has been shown to suppress pro-inflammatory cytokines [15–20], but not anti-inflammatory cytokines [6, 7], suggesting that ketamine may be an immunomodulatory rather than an immunosuppressive drug.

Furthermore, ketamine has immunomodulatory effects on nitric oxide (NO) production [7, 21, 22], macrophage and microglial function [17, 23, 24], neutrophil activation and migration [6, 25–28], and other immune cells, mediators, and pathways [29–33]. These studies demonstrated that ketamine has a wide range of effects on the immune system, thus highlighting the importance of determining the drug’s overall impact on inflammation for clinical use. However, experimental models using ketamine vary in terms of the route of drug administration and dosage, making it difficult to accurately characterize ketamine’s effects.

Nevertheless, the immunomodulatory properties of ketamine have been established experimentally in a variety of models. The bacterial endotoxin lipopolysaccharide (LPS) normally increases pro-inflammatory cytokines, such as interleukin (IL)-6, IL-1β, and TNF-α, but ketamine inhibited the production of these cytokines in vitro [24, 34]. In addition, rodent studies showed that LPS-induced systemic inflammation and neuroinflammation were attenuated by sub-anesthetic doses of ketamine [18, 22, 24]. Other rodent studies demonstrated that ketamine reduced TBI-induced production of inflammatory cytokines [35–37] and inflammatory cytokines in models of endotoxemia [38, 39]. These findings illustrate the immunomodulatory effects of ketamine in a variety of peripheral and central inflammatory models.

However, potential sex-related differences in the effects of ketamine on inflammatory markers are not well characterized. Preclinical studies have demonstrated that female rodents exhibit greater sensitivity to the antidepressant effects of ketamine than males [40, 41]. Ketamine also produces sex-related differences in dissociative stereotypy and antinociception [42] and pharmacokinetics [43] in rats; female rats metabolize ketamine more slowly than males, leading to a higher sustained drug concentration over time and contributing to greater behavioral effects. Moreover, males and females may have differential cytokine expression profiles in non-inflammatory [44–46] and inflammatory [47, 48] conditions, subsequently influencing behavioral differences [47, 49, 50]. Because the majority of preclinical studies have examined ketamine’s effects in male animals only, the current study investigated the effects of sub-anesthetic doses of ketamine on inflammatory cytokines in both male and female rats. This is a significant contribution to the existing body of literature due to the growing evidence that males and females may demonstrate different immune responses [47, 51]. In addition, the present study determined the effects of IV ketamine in a non-inflammatory condition, which has not been well characterized previously. Thus, examining non-inflammatory conditions may help characterize ketamine’s effects on immune function without a specific type of inflammatory stimulus. This may also provide greater knowledge for future studies aiming to understand potential therapeutic effects of ketamine on a variety of inflammatory disorders and conditions.

The current study used an IV ketamine infusion, which is the preferred route of administration in humans [1], to enhance clinical relevance and translation. In contrast, a majority of preclinical studies examining the use of ketamine and its immunomodulatory effects used a bolus intraperitoneal (IP) injection of ketamine, which is different from IV ketamine administration in a clinical setting. To our knowledge, this is the first study that investigated plasma cytokine levels over time following an IV ketamine infusion in rats, which is important for clinical translation. Specifically, ketamine was administered through an indwelling jugular venous catheter in awake and freely moving rats. The catheter also enabled repeated blood sampling after infusion as described previously [42, 52, 53]. Importantly, this technique does not require anesthesia or restraint of animals as compared to other preclinical methods of blood collection. This study investigated the effects of a single IV ketamine infusion on plasma cytokine levels in male and female rats. Either a sub-dissociative (10 mg/kg) or dissociative (40 mg/kg) dose was infused intravenously over a 2-h period, during which spontaneous locomotor activity was also monitored. Finally, to determine the effects of circulating estrogen levels on ketamine effects, the estrous cycle was monitored in female rats. We hypothesized that an IV ketamine infusion may reduce inflammatory cytokine levels in male and female rats, and that female rats may be more sensitive to ketamine.

## Materials and methods

### Animals

Adult Sprague–Dawley rats (9 weeks old, Envigo Laboratories, Dublin, VA), with male rats weighing 300–350 g and female rats weighing 200–250 g upon arrival, were individually housed in transparent Plexiglass shoebox cages in a climate-controlled environment. Animals were on a reversed 12-h light/dark cycle (light off at 0600; light on at 1800) with food and water available ad libitum. All procedures were performed in accordance with the National Institutes of Health *Guide for the Care and Use of Laboratory Animals* and were approved by the Institutional Animal Care and Use Committee (IACUC) at the Uniformed Services University (Bethesda, MD).

### Catheter surgery

Rats were anesthetized with isoflurane and a jugular venous catheter was implanted as described previously [54, 55]. In summary, the right jugular vein was isolated via a neck incision, and a polyurethane catheter (Instech, Plymouth Meeting, PA) was inserted into the vein. The catheter port exited through a back incision. The external portion of the catheter was made with a blunt tip 22-gauge stainless steel cannula cemented into place with bell-shaped dental acrylic. All catheters were flushed every 3 days to maintain patency and locked with 0.1 mL of sterile heparin and glycerol solution (1:1 ratio). Animals were given a minimum of 1 week to recover from surgery prior to the experiment and remained ketamine naive.

### Estrous cycle monitoring

Female rat estrous cycle was monitored for 2 weeks (5–6 days per week) as described previously [42] using a vaginal cytology method to obtain a baseline data. Briefly, vaginal cells were collected using a vaginal swab, spread onto microscope slides (Fisher Scientific, Waltham, MA), and dried. Smears were then stained with 0.02% crystal violet staining solution (Sigma-Aldrich, St. Louis, MO) to determine cellular morphology. Based on the predominant cell type on the day of experiment, rats were classified into either low (diestrus phase with mainly leukocytes) or a high (proestrus or estrus phases with mainly nucleated and/or cornified epithelial cells) estrogen group as described previously [42].

### Intravenous ketamine infusion and blood sampling

**Fig. 1:**
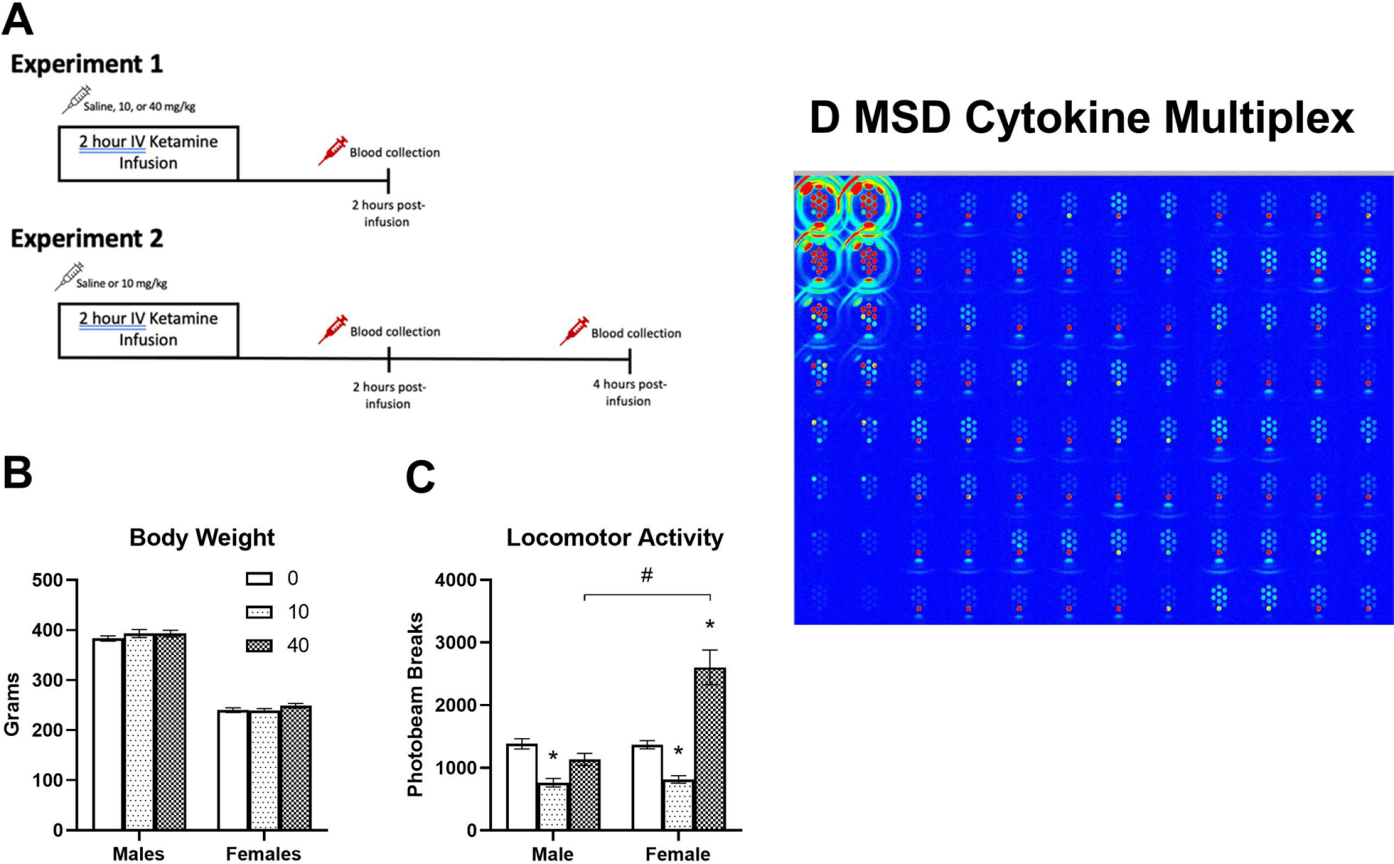
Overall study design and locomotor activity of male and female rats during an IV ketamine infusion. **A** Study design of Experiments 1 and 2. **B** Average body weights of adult male and female rats used in this study. **C** Spontaneous locomotor activity monitored by infrared photobeams during the 2-h infusion period. 10 mg/kg ketamine reduced activity both in male and female rats, but 40 mg/kg ketamine stimulated activity in female rats only. **D** A representative image of MSD cytokine multiplex assay. The first two columns on the left indicate a serial dilution of standards (S1 to S8) and the rest of columns indicate plasma samples loaded in the 96-well assay plate. Individual spots in each well indicate different cytokines. The sample sizes for body weight and locomotor activity: Saline controls (*n* = 25 for males and *n* = 25 for females), ketamine 10 mg/kg (*n* = 19 for males and *n* = 25 for females), and ketamine 40 mg/kg (*n* = 18 for males and *n* = 9 for females). *Significant from the controls (*p* < 0.05), #Significant between male and female rats (*p* < 0.05)

Racemic ( ±) ketamine hydrochloride (100 mg/mL) (Henry Schein Animal Health, Dublin, OH) was diluted in 0.9% sterile saline prior to the infusion. For Experiment 1 (dose–response testing), animals received a single saline or ketamine infusion (0, 10, or 40 mg/kg, IV) over a 2-h period. For Experiment 2 (time course testing), a within-subjects repeated measures design was used so that each animal received either saline or 10 mg/kg ketamine in a counter-balanced design with a 1-week interval between doses. Animals were given IV bolus saline or ketamine doses prior to placement into the infusion chambers (Med Associates Inc., St. Albans, VT) for the remainder of infusion. Each chamber (14″ L × 12″ W × 15″ H) was equipped with an infusion pump (Harvard Pump 11 Elite, Holliston, MA) using a 5 mL plastic syringe connected to a fluid swivel (Instech, Plymouth Meeting, MA) by polyurethane tubing encased in a metal spring-wire tether. The metal tether was attached to the metal cannula on the exit portion of the catheter between the rat scapulae using either a Luer-lock combination or a threaded swivel. The tether allowed free movement of the animals in the chamber during the infusion. Chambers were equipped with two infrared photobeams that measured the spontaneous horizontal activity of animals throughout the ketamine infusion. A computer recorded the number of photobeam breaks to record locomotor activity levels. An overall study design is illustrated in Fig. 1A. For Experiment 1 (dose–response testing), animals were euthanized and trunk blood samples were collected at 2-h post-ketamine infusion (0, 10, or 40 mg/kg, IV). For Experiment 2 (time course testing), catheter blood (approximately 0.3 mL) was collected from the animals at 2- and 4-h post-ketamine infusion (0 or 10 mg/kg, IV). Blood was collected in microfuge tubes containing 2.5 μL EDTA and centrifuged at 4,000 rpm for 10 min at 4 °C. Plasma was aliquoted into tubes and frozen at -40 °C.

### Cytokine multiplex immunoassay

To determine plasma inflammatory cytokine levels, an electrochemiluminescent multiplex immunoassay was used (Pro-inflammatory Rat Panel 2 V-PLEX, Meso Scale Discovery [MSD], Rockville, MD). The kit measures levels of the inflammatory cytokines IFN-γ, IL-1β, IL-4, IL-5, IL-6, KC/GRO, IL-10, IL-13, and TNF-α in the sample. Briefly, the MSD 96-well plate was blocked for 1 h, then standards and 4 × diluted samples (50 μL per well) were added in duplicate to the plate that was pre-coated with primary capture antibody for each cytokine. Samples were incubated at room temperature for 2 h shaking at 500 rpm. The plate was washed between each step with wash buffer (10 × PBS with 0.05% Tween 20). Secondary detection antibodies for each cytokine were then added and the plate was incubated at room temperature for 2 h shaking at 500 rpm. After washing, a read buffer was added, and the plate was then read on the MSD Sector Image 6000 (Meso Scale Discovery, Rockville, MD) using the corresponding Workbench software. The lower limit of detection for each cytokine antibody was 0.65 pg/mL (IFN-γ), 6.92 pg/mL (IL-1β), 0.69 pg/mL (IL-4), 14.1 pg/mL (IL-5), 13.8 pg/mL (IL-6), 1.04 pg/mL (KC/GRO), 16.4 pg/mL (IL-10), 1.97 pg/mL (IL-13), and 0.72 pg/mL (TNF-α). A representative image of the MSD cytokine multiplex assay is shown in Fig. 1D. The first two columns on the left display a serial dilution of standards (S1 to S8) and the rest of the columns show plasma samples loaded in the 96-well assay plate. Individual spots in each well indicate different cytokines.

### Statistical analysis

All data are presented as mean ± standard error of the mean (SEM). Locomotor activity and cytokines are reported as changes relative to the saline control group to control for variation across plates. Data were analyzed using GraphPad Prism (GraphPad Software, version 9.0). Significance was determined through *p* values < 0.05. For the dose–response data, a two-way ANOVA with sex and ketamine as independent variables was used with Holm–Sidak multiple comparisons tests. For the time course data, a three-way ANOVA with sex, ketamine, and time as independent variables was used with Holm–Sidak tests for multiple comparisons. Three cytokines (IFN-γ, IL-1β, and IL-13) in plasma samples were below the detection limit, and therefore, data are not reported. Among the rest of the cytokines analyzed, there were missing values, especially for IL-4, IL-5, and IL-6, and thus the total number of samples included in the analysis varied across groups. Specific numbers of samples included in each analysis can be found in the figure legends.

## Results

Average body weights of male and female rats are shown in Fig. 1B. Although male rats were significantly larger than female rats, there were no differences between the saline control, 10, and 40 mg/kg groups. A single ketamine infusion produced dose-dependent effects on spontaneous locomotor activity in male and female rats during the infusion (Fig. 1C). In male rats, 10 mg/kg ketamine reduced locomotor activity. However, in female rats, 10 mg/kg ketamine reduced activity and 40 mg/kg ketamine stimulated activity. A two-way ANOVA indicated significant main effects of ketamine F (2, 115) = 59.3, *p* < 0.001 and sex F (1, 115) = 41.34, *p* < 0.001, and an interaction F (2, 115) = 31.18, *p* < 0.001 on locomotor activity. Overall, the sub-anesthetic IV ketamine infusion produced dose-dependent and sex-specific effects on locomotor activity in adult male and female rats.

**Fig. 2:**
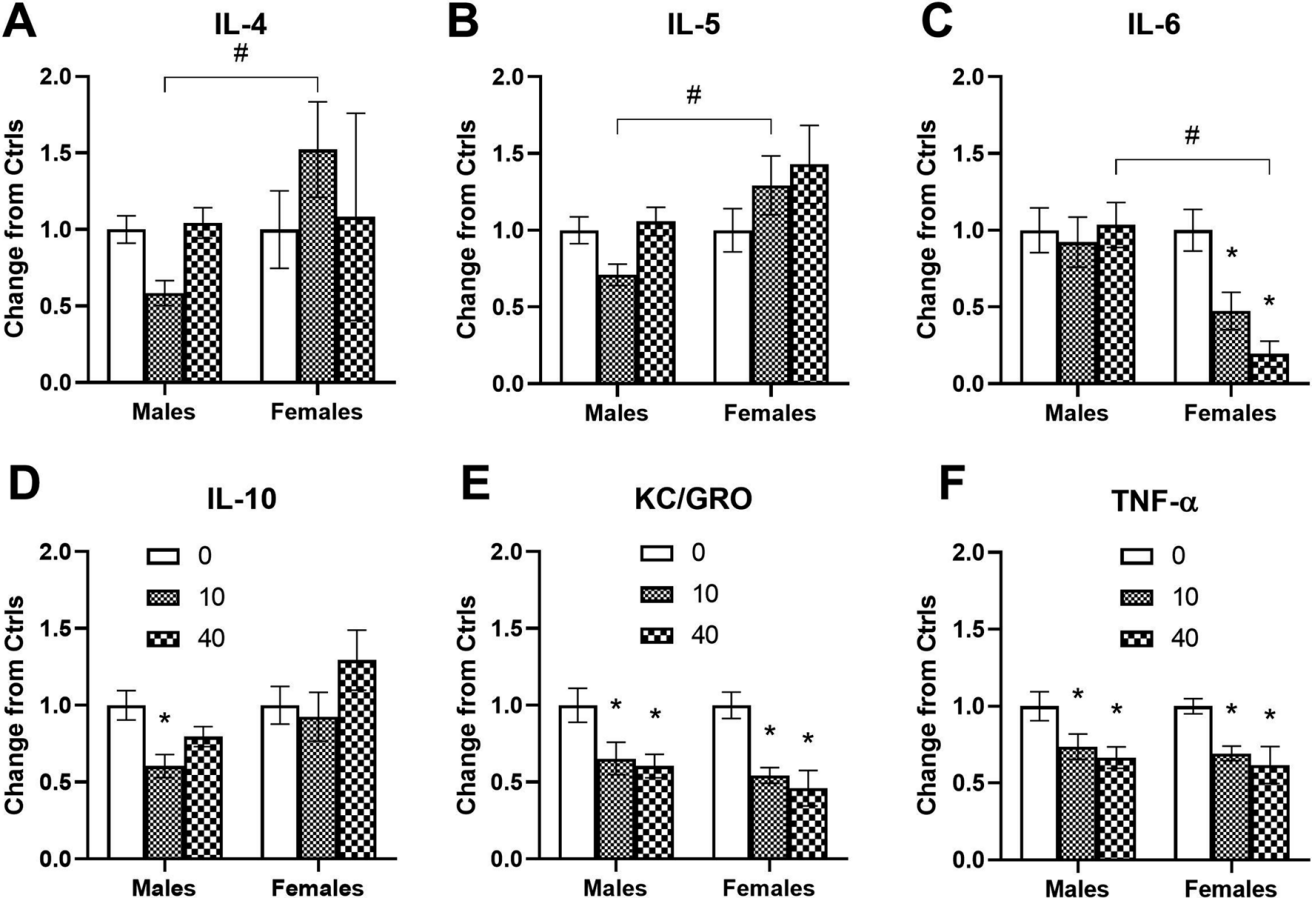
Dose-dependent and sex-specific effects of IV ketamine infusion on plasma cytokine levels in male and female rats. Six different cytokine levels at 2-h post-infusion of ketamine (0, 10 or 40 mg/kg) in male and female rats are shown. Data are presented as changes from the saline control group to reduce variations in absolute values of cytokines between different assay plates. **A** IL-4 levels are significantly different between male and female rats following 10 mg/kg ketamine infusion. **B** IL-5 levels are significantly different between male and female rats following 10 mg/kg ketamine infusion. **C** IL-6 levels are significantly reduced in female rats following ketamine infusion (10 and 40 mg/kg). **D** IL-10 levels are significantly reduced in male rats following 10 mg/kg ketamine infusion. **E** KC/GRO levels are reduced in male and female rats following ketamine infusion (10 and 40 mg/kg). **F** TNF-α levels are reduced in male and female rats following ketamine infusion (10 and 40 mg/kg). The sample sizes for cytokines (saline, 10, and 40 mg/kg ketamine): IL-4 (*n* = 23, 18, 17 for males and *n* = 11, 11, 4 for females), IL-5 (*n* = 24, 18, 17 for males and *n* = 14, 9, 5 for females), IL-6 (*n* = 25, 19, 18 for males and *n* = 19, 15, 7 for females), IL-10 (*n* = 25, 18, 17 for males and *n* = 18, 16, 9 for females), KC/GRO (*n* = 24, 18, 16 for males and *n* = 25, 24, 9 for females), and TNF-α (*n* = 25, 19, 18 for males and *n* = 25, 25, 8 for females). *Significant from the controls (*p* < 0.05), #Significant between male and female rats (*p* < 0.05)

The ketamine infusion also produced dose-dependent and sex-specific effects on plasma cytokine levels when measured at 2-h post-infusion (Fig. 2). A two-way ANOVA indicated a significant interaction between ketamine and sex F (2, 78) = 4.361, *p* = 0.016 on IL-4 levels. Post hoc tests revealed significant differences between male and female rats following a 10 mg/kg ketamine infusion (*p* < 0.05).

Figure 2B shows the effects of ketamine on IL-5 levels in male and female rats. A two-way ANOVA indicated a significant main effect of sex F (1, 81) = 8.792, *p* = 0.004 and an interaction between ketamine and sex F (2, 81) = 3.306, *p* = 0.042. Post hoc tests revealed significant differences between male and female rats following a 10 mg/kg ketamine infusion.

Both 10 mg/kg and 40 mg/kg ketamine reduced plasma IL-6 levels in female rats (Fig. 2C). A two-way ANOVA indicated significant main effects of ketamine F (2, 97) = 3.503, *p* = 0.034 and sex F (1, 97) = 10.3, *p* = 0.002, and an interaction F (2, 97) = 3.317, *p* = 0.04. Post hoc tests revealed that both 10 mg/kg and 40 mg/kg doses significantly reduced IL-6 levels in female rats (*p* < 0.05). There was a significant difference between male and female rats following a 40 mg/kg ketamine infusion (*p* < 0.05).

In contrast, IL-10 levels were reduced in male rats following a 10 mg/kg ketamine infusion (Fig. 2D). A two-way ANOVA indicated significant main effects of ketamine F (2, 97) = 3.211, *p* = 0.045 and sex F (1, 97) = 7.763, *p* = 0.006. Post hoc tests revealed that 10 mg/kg ketamine significantly reduced IL-10 levels in male rats (*p* < 0.05).

Figure 2E illustrates that plasma KC/GRO levels were significantly reduced in both male and female rats following 10 and 40 mg/kg ketamine infusions. A two-way ANOVA indicated that there was a significant main effect of ketamine F (2, 110) = 15.26, *p* < 0.001 on KC/GRO levels. Post hoc tests revealed that both 10 and 40 mg/kg doses significantly reduced KC/GRO levels both in male and female rats (*p* < 0.05).

Similar to KC/GRO, a ketamine infusion also reduced TNF-α levels in both male and female rats (Fig. 2F). A two-way ANOVA indicated a significant main effect of ketamine F (2, 114) = 12.46, *p* < 0.001 on TNF-α levels. Post hoc tests revealed that both 10 and 40 mg/kg ketamine doses significantly reduced TNF-α levels in male and female rats (*p* < 0.05).

**Fig. 3:**
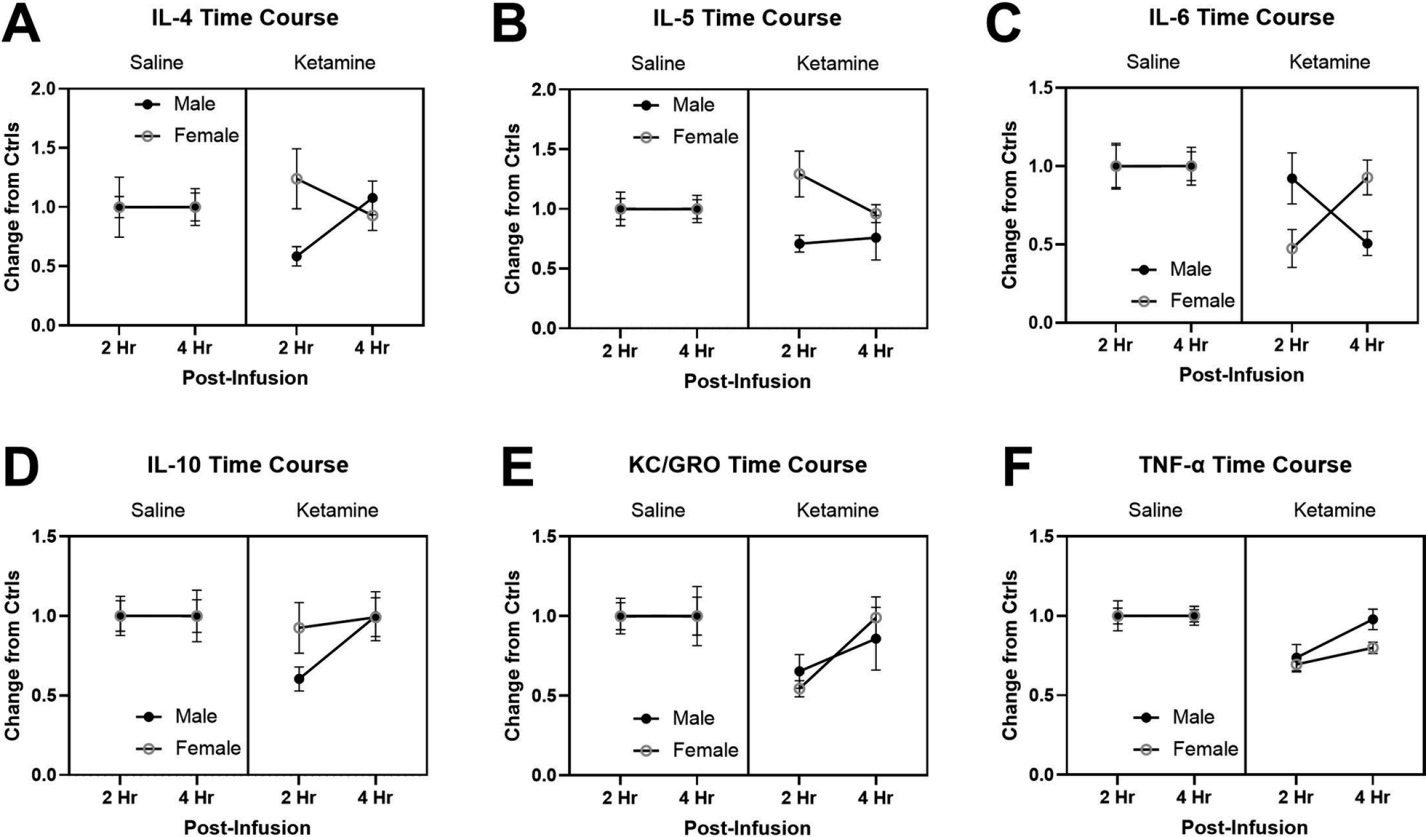
Time course of plasma cytokine levels at 2- and 4-h post-ketamine infusion (0 or 10 mg/kg) in male and female rats. Data are presented as changes from the saline control group. The saline infusion did not change any of the cytokine levels at 2- and 4-h post-infusion in male and female rats. However, ketamine-induced reduction of cytokine levels at 2-h post-infusion returned to the control levels at 4-h post-infusion, except for IL-6 levels in male rats. This indicates that a 10 mg/kg ketamine infusion produces a delayed reduction of IL-6 levels in male rats only (3C). The sample sizes for cytokines (2 h and 4 h): IL-4 (*n* = 23, 21 for saline males and *n* = 11, 15 for saline females; *n* = 18, 19 for ketamine males and *n* = 11, 10 for ketamine females), IL-5 (*n* = 24, 12 for saline males and *n* = 14, 14 for saline females; *n* = 18, 15 for ketamine males and *n* = 9, 13 for ketamine females), IL-6 (*n* = 25, 13 for saline males and *n* = 19, 17 for saline females; *n* = 19, 18 for ketamine males and *n* = 15, 16 for ketamine females), IL-10 (*n* = 25, 21 for saline males and *n* = 18, 20 for saline females; *n* = 18, 19 for ketamine males and *n* = 16, 16 for ketamine females), KC/GRO (*n* = 24, 20 for saline males and *n* = 25, 21 for saline females; *n* = 18, 18 for ketamine males and *n* = 24, 21 for ketamine females), TNF-α (*n* = 25, 21 for saline males and *n* = 25, 21 for saline females; *n* = 19, 19 for ketamine males and *n* = 25, 22 for ketamine females)

In addition to the ketamine dose–response test, a time course of 10 mg/kg ketamine on cytokine levels was determined at 2- and 4-h post-infusion in male and female rats. Figure 3A shows a time course of IL-4 levels in male and female rats following ketamine administration. A three-way ANOVA revealed non-significant interactions between sex and time F (1, 120) = 3.36, *p* = 0.069, and ketamine, sex, and time F (1,120) = 3.397, *p* = 0.067 on IL-4 levels. The IL-4 levels returned to control levels at 4-h post-ketamine infusion.

Figure 3B shows the effects of ketamine on plasma IL-5 levels at 2- and 4-h post-infusion in rats. A three-way ANOVA indicated a significant main effect of sex F (1, 111) = 5.133, *p* = 0.025 and an interaction between ketamine and sex F (1, 111) = 5.171, *p* = 0.025 on IL-5 levels. Post hoc tests revealed a significant difference in IL-5 levels between male and female rats at 2-h post-infusion (*p* < 0.05).

Compared to IL-4 and IL-5 levels, IL-6 levels showed a different trajectory over time following the ketamine infusion (Fig. 3C). A three-way ANOVA indicated a significant main effect of ketamine F (1, 134) = 9.506, *p* = 0.003, and an interaction between time and sex F (1, 134) = 5.264, *p* = 0.023 as well as an interaction between ketamine, sex, and time F (1, 134) = 5.264, *p* = 0.023. The IL-6 levels in male rats were decreased at 4-h post-infusion, whereas reduced IL-6 levels in female rats returned to control levels at 4-h post-infusion.

The effects of ketamine on the time course of IL-10 levels in male and female rats are shown in Fig. 3D. A three-way ANOVA indicated that there was no significant main effect or interaction between ketamine, sex, and time on IL-10 levels.

The time course of KC/GRO levels between male and female rats is shown in Fig. 3E. A three-way ANOVA indicated a significant main effect of ketamine F (1, 163) = 7.216, *p* = 0.008 on KC/GRO levels. The KC/GRO levels were reduced at 2-h post-infusion and returned to control levels at 4-h post-infusion in both in male and female rats.

Similarly, ketamine-induced reduction of TNF-α levels at 2-h post-infusion was recovered by 4-h post-infusion in male rats (Fig. 3F). A three-way ANOVA indicated significant main effects of ketamine F (1, 169) = 19.77, *p* < 0.001 and time F (1, 169) = 3.81, *p* = 0.005, and an interaction between ketamine and time F (1, 169) = 3.755, *p* = 0.05. Post hoc tests revealed significant differences in TNF-α levels between 2-h saline and ketamine samples collected from both male and female rats.

**Fig. 4:**
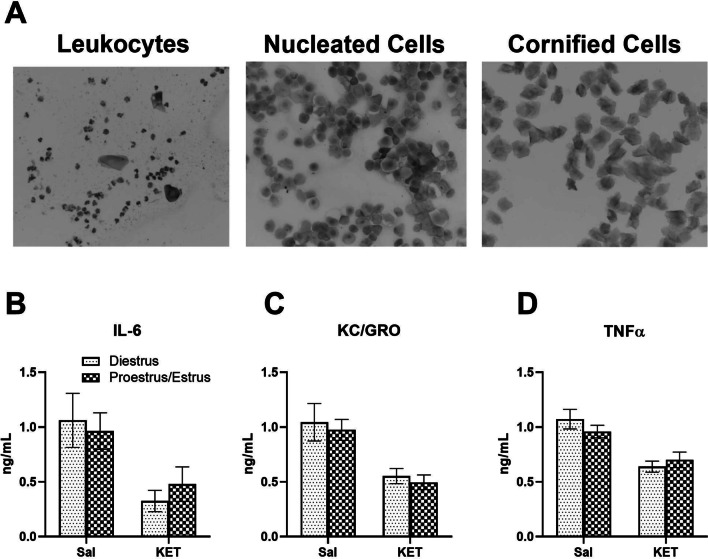
No effects of estrous cycle on IV ketamine-induced reduction of plasma IL-6, KC/GRO, and TNF-α levels in female rats. **A** Different vaginal cell types indicating different phases of estrous cycle in female rats: leukocytes (diestrus phase), nucleated epithelial cells (proestrus phase), and cornified epithelial cells (estrus phase). Ketamine infusions reduced IL-6 (**B**), KC/GRO (**C**), and TNF-α (**D**) levels as compared to the saline controls, but there were no significant differences between the diestrus (low estrogen levels) and proestrus/estrus (high estrogen levels) phases on these cytokine levels. The sample sizes for cytokines (diestrus and proestrus/estrus phases): IL-6 (*n* = 7, 12 for saline females and *n* = 7, 15 for ketamine females), KC/GRO (*n* = 9, 16 for saline females and *n* = 14, 19 for ketamine females), and TNF-α (*n* = 9, 16 for saline females and *n* = 14, 19 for ketamine females)

Because ketamine reduced three cytokine levels in female rats, these cytokine (IL-6, KC/GRO, and TNF-α) levels were further analyzed using the estrous cycle data collected from the female rats (Fig. 4). Based on the major vaginal cell types determined on the day of ketamine testing, female rats were classified as either low estrogen group (diestrus phase) or high estrogen group (proestrus and estrus phases). As both 10 mg/kg and 40 mg/kg doses reduced IL-6, KC/GRO, and TNF-α levels, data from the two doses were combined for this analysis. Although a ketamine infusion reduced these cytokine levels in female rats as compared to male rats, there were no significant differences between the low and high estrogen groups.

A two-way ANOVA indicated a significant main effect of ketamine F (1, 37) = 10.69, *p* = 0.002 on IL-6 levels (Fig. 4B). However, post hoc tests on IL-6 levels revealed no significant differences between the diestrus and proestrus/estrus groups. A two-way ANOVA indicated a significant main effect of ketamine F (1, 54) = 25.45, *p* < 0.001 on KC/GRO levels (Fig. 4C). Post hoc tests on KC/GRO levels revealed no significant differences between the diestrus and proestrus/estrus groups. A two-way ANOVA indicated a significant main effect of ketamine F (1, 54) = 24.51, *p* < 0.001 on TNF-α levels (Fig. 4D). Post hoc tests on TNF-α levels revealed no significant differences between the diestrus and proestrus/estrus groups. These results indicate that the immunomodulatory effects of IV ketamine infusion on these inflammatory cytokines are independent of endogenous estrogen levels in female rats.

## Discussion

The current study investigated the effects of sub-anesthetic doses of an IV ketamine infusion (0, 10, or 40 mg/kg, IV) on inflammatory cytokine levels in male and female Sprague–Dawley rats. The time course of cytokine changes following the ketamine infusion (0 or 10 mg/kg, IV) were further determined at 2- and 4-h post-infusion. The effects of the estrous cycle on ketamine-induced cytokine changes were also analyzed in female rats. Overall, the IV ketamine infusion produced dose-dependent and sex-specific changes on multiple cytokine levels in male and female Sprague–Dawley rats. To our knowledge, this is the first study to examine the effects of a subanesthetic IV ketamine infusion on inflammatory cytokine levels in male and female rats. The results shown here contribute to the understanding of sex-specific effects of IV ketamine and whether the estrous cycle affects these outcomes. In addition, examining a non-inflammatory condition helps build foundational knowledge on ketamine’s basal influence on the immune response and allows for clinical translation to a wide variety of inflammatory disorders and conditions.

The 10 mg/kg ketamine infusion decreased locomotor activity in male and female rats, due to sedative effects at this dose. However, 40 mg/kg ketamine (stronger analgesic but dissociative) led to increased locomotor activity in female rats, indicating that female rats may be more sensitive to the dissociative effects of ketamine. This finding is consistent with previous studies reporting greater sensitivity to ketamine’s effects on locomotor activity in female rodents [56, 57]. It is possible that ketamine-induced locomotor activity stimulation in female rats could be due to enhanced dissociative stereotypy, such as ataxia, when compared to that of male rats [42]. In addition, sex differences in the pharmacokinetic or monoaminergic activity of ketamine [58] may have contributed to enhanced locomotor stimulation in female rats. These results demonstrate that female rats exhibit greater sensitivity to locomotor stimulatory effects of ketamine at dissociative doses.

Following an IV ketamine infusion (0, 10, or 40 mg/kg), plasma cytokine levels were determined at 2-h post-infusion in male and female rats. IL-10 is an anti-inflammatory cytokine that plays an essential role in immune regulation by inhibiting the production of pro-inflammatory cytokines [59]. Interestingly, a 10 mg/kg ketamine infusion reduced IL-10 levels in male rats. Similar to these findings, a previous study reported that a single injection of ketamine (7 mg/kg, IP) after an LPS stimulus attenuated blood IL-10 levels in male rats, though this was observed 5-h post-injection [35]. Thus, it is possible that lower doses of ketamine (< 10 mg/kg) administration produce sex-specific effects on IL-10 levels, and this should be further investigated to validate the immunomodulatory effects of ketamine.

In contrast, both 10 mg/kg and 40 mg/kg ketamine reduced pro-inflammatory cytokines including IL-6, KC/GRO, and TNF-α in rats. IL-6 is an important pro-inflammatory cytokine that is involved in the acute immune response after a pathogen exposure and plays a role in neutrophil attraction, B and T cell responses, metabolism, pain, and neural functions which are often associated with inflammatory and autoimmune disorders [44, 60]. KC/GRO, the rodent equivalent of CXCL1, is a chemokine that attracts neutrophils to the site of inflammation [61, 62]. TNF-α is an important pro-inflammatory cytokine that has widespread effects on the immune response, apoptosis and necrosis, and is associated with neurodegenerative diseases [63].

Previous studies have reported that ketamine reduced both IL-6 and TNF-α levels. For example, ketamine reduced IL-6 levels in the blood of both male and female patients when added to human blood samples (500 μg/mL dose) [64] or administered to patients (0.15–0.25 mg/kg, IV) [64–66]. Other studies have shown that sub-anesthetic ketamine inhibited TNF-α production in multiple models and conditions, including in a rat model of endotoxin stimulus [67], human subjects with clinical depression [68], and LPS-induced inflammation in canines [69]. To our knowledge, no previous studies have examined KC/GRO after administration of a sub-anesthetic IV ketamine infusion. The dose-dependent effects of ketamine on pro-inflammatory cytokines have important implications for clinical use, including the treatment or prevention of injuries mediated by inflammation.

The immunomodulatory effects of low-dose ketamine have also been demonstrated in clinical surgical cases. Patients who were administered 0.15 mg/kg IV ketamine immediately before undergoing elective abdominal surgery had lower blood serum levels of IL-6 and TNF-α 4 h after surgery [66]. In another study, patients who were administered 0.25 mg/kg IV ketamine during cardiopulmonary bypass surgery exhibited reduced serum IL-6 levels immediately, 4 h, and up to 7 days after the procedure [65]. In fact, a meta-analysis of multiple studies determined that intraoperative ketamine decreases blood IL-6 levels throughout the postoperative period [70]. These findings are consistent with the current study demonstrating that sub-anesthetic doses of ketamine reduce pro-inflammatory cytokines in male and female rats.

It has been shown that a 10 mg/kg ketamine infusion over a 2-h period produces analgesic effects without dissociative symptoms in rats [55]. Because of this behavioral profile, this dose is clinically equivalent to the dose that is recommended for service members injured on the battlefield [3]. Low analgesic doses of ketamine appear to be highly favorable clinically, given such doses may possess immunomodulatory benefits without adverse dissociative effects [6]. Therefore, a 10 mg/kg IV ketamine dose was chosen for the time course analysis of cytokine changes (Experiment 2).

The time course of cytokine changes after a 10 mg/kg IV ketamine infusion revealed that ketamine’s effects are generally transient, leading to greater cytokine reductions at 2-h rather than at 4-h post-ketamine infusion. Interestingly, current results show sex-specific differences in the temporal fluctuations of IL-6 and TNF-α levels. Previous studies that have examined these cytokines as biomarkers have used varying timepoints. Thus, our findings of more transient effects of KC/GRO and IL-10 at 2-h and sex-specific temporal differences in IL-6 and TNF-α may hold important implications for the timing of ketamine’s immunomodulatory effects. It is also important to consider how the timing of cytokine changes induced by ketamine may be altered in different inflammatory states.

The present study demonstrated that male and female rats exhibit different cytokine expression profiles after ketamine exposure. This supports our hypothesis that sex-related differences exist given previous literature showing cytokine divergence based on biological sex. Our results indicate that ketamine reduced IL-6 levels 2-h post-infusion in female animals only. This is contrary to clinical studies reporting that ketamine reduced IL-6 blood concentrations in both male and female patients [64–66]. However, some studies reported that women have greater IL-6 expression levels than those of men, which is thought to be associated with the higher prevalence of autoimmune disorders among women [44]. Likewise, greater IL-6 production in female pigs has been linked to higher stress responses compared to those of male pigs [71]. In addition, a study found that the timing of peak IL-6 blood levels is also different based on sex, with men showing an earlier IL-6 peak than that of women during a stress response [72]. Our results also demonstrate sex-specific temporal differences in IL-6 levels. This heterogeneity of findings warrants further investigation on how biological sex differences may influence ketamine’s effect on IL-6 levels.

Both 10 and 40 mg/kg IV ketamine doses reduced KC/GRO levels in male and female animals. The temporal reduction of KC/GRO also appeared to be similar between male and female rats. This contrasts a study on hippocampal samples in mice after LPS that reported a more robust yet more transient increase in KC/GRO levels in female mice than in male mice [48]. However, another study found that male sex hormones led to the upregulation of CXCL1 in humans and KC/GRO in murine models [73]. Thus, future studies are warranted to further examine the clinical implications of ketamine effects on KC/GRO and how such interaction may be influenced by sex differences.

Both 10 mg/kg and 40 mg/kg IV ketamine reduced TNF-α in male and female rats. Ketamine’s reduction of TNF-α levels has been demonstrated in other studies. A study examining postoperative inflammation reported attenuated TNF-α levels in both male and female patients who received preoperative ketamine [66], and a study examining depressed patients [68] also showed similar results in both men and women. Consistent with our data, it appears that ketamine dose-dependently inhibits blood TNF-α levels in both sexes.

Sex differences in certain cytokine responses could be due to multiple physiological differences between males and females. Though the specific effects of biological sex on the immune response are still largely unknown, studies have shown that females across species generally show greater innate and adaptive immune responses [51]. In the present study, we monitored female estrous cycles to determine the influence of sex hormones on ketamine-induced cytokine changes. Investigating sex-related differences in drug effects is important for clinical translation. However, we found that ketamine’s effects on cytokine profiles were similar between female rats with high and low estrogen levels. This suggests that ketamine’s differential effects on cytokine levels in females compared to males could be due to innate basal differences between the sexes rather than the fluctuation of estrogen levels. Studies have shown that estrogen generally enhances cytokine secretion, while androgens decrease it [74]. Therefore, due to the expression of estrogen receptors on pro-inflammatory cells, females may exhibit a stronger immune response to an antigenic challenge, while males have increased susceptibility to infection due to androgen-mediated suppression of adaptive immune responses [73]. Though we did not see differences in cytokine levels between high- and low-estrogen females, sex hormone levels of males (androgen) and females (estrogen) may have influenced overall cytokine changes observed after ketamine administration.

Ketamine’s effect on cytokine levels can be attributed to several different mechanisms. Its anti-inflammatory properties can be attributed to the suppression of pro-inflammatory macrophage/microglia activation [17, 70]. Ketamine suppresses macrophage functions, such as phagocytosis and the production of inflammatory cytokines [17], potentially by reducing mitochondrial membrane potential [17] or suppressing toll-like receptors (TLRs) [75]. Ketamine’s effects on inflammatory T-cells, nitric oxide production, and numerous ion channels and receptors may also be associated with its anti-inflammatory properties [7]. Ketamine also reduces Ca^2+^ levels and inhibits CaMKII phosphorylation through NMDA blockade, and thus inhibits NF-κB translocation [76]. By interfering with both pro- and anti-inflammatory cytokine production, ketamine may act to realign the immune system to homeostasis following an inflammatory insult [6].

The immune system is a complex balance of immune modulators, and our data show that ketamine affected both pro- and anti-inflammatory cytokines. However, there is no clear consensus regarding ketamine’s effects on pro- vs anti-inflammatory cytokines. A study of patients with major depression found that both pro-and anti-inflammatory cytokines were downregulated after repeated ketamine administration [77]. In contrast, a review of ketamine’s anti-inflammatory effects found it to reduce pro- but not anti-inflammatory cytokines [6]. A later review by this group suggested that ketamine may function more as a homeostatic regulator of the immune system, promoting balance of both pro- and anti-inflammatory elements, as opposed to an immunosuppressant [7]. Our data support an immunological balance effect as evidenced by ketamine’s reduction of not only pro-inflammatory cytokines but also, interestingly, the anti-inflammatory cytokine IL-10. However, our data also show that these effects are sex-specific. More studies are needed to understand the complex effects of ketamine on the immune response, especially as the present study demonstrates that these effects could vary based on sex.

The present study was performed on rats in a non-inflammatory condition, without any inflammatory stimulus. Most studies examining the immunomodulatory properties of ketamine are completed in an inflammatory condition, such as LPS, surgery, injury, or depression. In fact, several reviews argue that ketamine may have no effect on cytokines unless in the presence of a stress-induced increase of inflammation [6, 7]. Our results dispute the requirement for an inflammatory state, showing that IV ketamine can reduce plasma cytokine levels in animals that were not subjected to an inflammatory stimulus. However, it is unknown whether analgesic doses of an IV ketamine infusion also reduce plasma cytokine levels in animals with an inflammatory condition. Therefore, future studies should investigate ketamine effects under those conditions.

A limitation of this study includes the low sensitivity for three cytokines (IFN-γ, IL-1β, and IL-13) using the MSD assay, which were not reported in this study due to excessive missing values among the samples analyzed. The lack of detection of these cytokines may have been due to the non-inflammatory condition of the animals in the current study. In addition, measuring cytokine levels at an earlier timepoint, such as 1-h post-ketamine infusion, could have further elucidated the peak of ketamine effects in these samples. Furthermore, the effects of IV ketamine on central inflammation in the brain and corresponding behaviors are largely unknown. It is thus important to consider ketamine’s effects in the context of a central inflammatory insult, such as a TBI. Males and females show different inflammatory profiles following TBI, and sex hormones can influence TBI outcomes [50]. Thus, it is important to understand the effects of ketamine on sex-related differences with respect to functional outcomes after an injury.

## Conclusions

Sub-anesthetic doses of an IV ketamine infusion produced immunomodulatory effects in male and female rats. The present study demonstrated ketamine-induced reductions in pro- and anti-inflammatory cytokines in a sex-specific manner. These results demonstrate the importance of studying both sexes when examining potential mechanisms of ketamine in preclinical studies. In addition, the use of an IV model that temporally characterizes cytokine expressions post-ketamine is clinically translational. The differential effects that IV ketamine may have on males and females provide important insight for clinical research and practice, ranging from analgesia to antidepressant treatment, and are important factors to consider when advancing toward a future approach of personalized medicine.

## Data Availability

Data generated and analyzed for this study are available from the corresponding author on reasonable request.
